# LncRNA SNHG4 Regulates Lipid Metabolism and Inflammation in Non-Alcoholic Fatty Liver Disease by Targeting miR-34b-5p/XIAP Axis

**DOI:** 10.5152/tjg.2025.24191

**Published:** 2025-05-05

**Authors:** YanJu Ding, Sheng Zheng, Xin Nian Fu, Chi Ma, Yang Juan

**Affiliations:** 1 Department of General Practice, Wuhan Third Hospital (Tongren Hospital of WuHan University), Hubei, China; 2 Department of Gastroenterology, The Third People’s Hospital of Yunnan Province, Yunnan, China; 3 Dali University Graduate School of Clinical Medicine, Yunnan, China; 4 Department of Science and Education, Affiliated Hospital of Yunnan University, Yunnan, China

**Keywords:** Inflammation, lipid metabolism, LncRNA SNHG4, miR-34b-5p, non-alcoholic fatty liver disease, XIAP

## Abstract

**Background/Aims:**

This research aimed at probing the mechanism of long non-coding RNA small nucleolar RNA host gene 4 (SNHG4) in regulating lipid metabolism and inflammation in non-alcoholic fatty liver disease (NAFLD).

**Materials and Methods:**

L02 and THLE-2 cells were stimulated with free fatty acids (FFA) and transfected. Lipid accumulation was detected through Oil Red O staining, measurements of triglyceride and total cholesterol were taken, and the levels of tumor necrosis factor-α, interleukin (IL)-1β, and IL-6 were assessed using enzyme-linked immunosorbent assays. The assessment of gene expression was conducted using real-time reverse transcriptase-polymerase chain reaction and Western blot techniques. The interplay between SNHG4 and miR-34b-5p/X-linked inhibitor of apoptosis protein (XIAP) was evaluated.

**Results:**

Free fatty acids downregulated SNHG4 and XIAP and upregulated miR-34b-5p in L02 and THLE-2 cells, leading to lipid metabolism disorder and inflammation. SNHG4 overexpression mitigated the lipid metabolism disorder and inflammation triggered by FFA, while this effect was suppressed by silencing XIAP. In contrast, SNHG4 knockdown aggravated FFA-induced lipid metabolic and inflammatory disorders, whilst this effect was rescued by inhibiting miR-34b-5p.

**Conclusion:**

SNHG4 regulates lipid metabolism and inflammatory disorders in NAFLD by targeting the miR-34b-5p/XIAP axis.

**Main Points**

- Overexpressing SNHG4 ameliorates lipid metabolism disorders and inflammation induced by free fatty acids.
- miR-34b-5p is involved in the regulation of lipid metabolism and inflammation in non-alcoholic fatty liver disease by SNHG4.
- SNHG4 ameliorates free fatty acids-induced lipid metabolism disorders and inflammation by miR-34b-5p/XIAP axis.

## Introduction

Non-alcoholic fatty liver disease (NAFLD) is a liver disease characterized by excessive accumulation of fat in hepatocytes in the absence of a significant history of alcohol consumption.^1^ The pathogenesis of NAFLD involves a variety of factors such as insulin resistance, lipid metabolism disorders, oxidative stress, and inflammatory responses.^2^^,^^3^ Non-alcoholic fatty liver disease ranges from simple steatosis (fatty liver) to non-alcoholic steatohepatitis (NASH), a condition that can then progress to liver cirrhosis, fibrosis, or even hepatocellular carcinoma.^4^ Currently, the treatment of NAFLD still lacks specific drugs and relies mainly on lifestyle interventions, such as weight loss, dietary control, and increased physical activity, which can ameliorate hepatic steatosis and inflammation.^5^ In recent years, the exploration of molecular mechanisms and biomarkers of NAFLD has remained a hotspot, especially in relation to intestinal flora, hepatic fibrotic progression, and genetic susceptibility. However, the etiology of NAFLD and its diverse pathologic processes complicate therapeutic strategies. Therefore, further exploration of the underlying molecular mechanisms of NAFLD pathogenesis is of great significance for future drug development and the formulation of new individualized therapeutic regimens.

The molecular mechanism of NAFLD pathogenesis has been extensively explored, in which RNA networks have been constructed to support effective diagnosis or treatment strategies for NAFLD.^6^^-^^8^ Accumulating lncRNAs have been discussed in NAFLD, such as lncRNA Metastasis Associated Lung Adenocarcinoma Transcript 1 (MALAT1)^9^ and lncRNA Nuclear Enriched Abundant Transcript 1 (NEAT1)^10^ in the regulation of hepatic lipogenesis, and lncRNA Zinc Finger NFX1-Type Containing 1 antisense RNA 1 (ZFAS1) in lipid peroxidation and inflammation.^11^ LncRNA small nucleolar RNA host gene 4 (SNHG4) is abnormally expressed in hepatocellular carcinoma, and its specific mechanism as a competing endogenous RNA has been extensively explored.^12^^,^^13^ However, the specific function of SNHG4 in NAFLD has been rarely studied.

LncRNAs acting as miRNA sponges have been shown to be key players in NAFLD pathogenesis in recent years.^14^ For example, LncRNA Gm28382 promotes NAFLD adipogenesis via the carbohydrate-responsive element-binding protein signaling pathway through miR-326-3p.^15^ In addition, histone H3 lysine 27 acetylation activates NEAT1, which promotes hepatic lipid accumulation in NAFLD by regulating the miR-212-5p/glutamate receptor subunit 3 gene.^16^ miR-34b-5p is found to promote inflammation in diseases.^17^^,^^18^ There is no clear indication of whether miR-34b-5p plays a role in NAFLD-induced inflammation.

X-linked inhibitor of apoptosis protein (XIAP) is a member of apoptosis inhibitory proteins, which has become one of the hotspots of biological research in recent years due to its key role in apoptosis inhibition.^19^ X-linked inhibitor of apoptosis protein is encoded by the BIRC4 gene, which is located on the X chromosome, and its main function is to inhibit the apoptosis effector proteins by directly binding to the key apoptosis effector proteins, thereby inhibiting apoptosis. Several works have demonstrated that XIAP plays a role in NAFLD. For example, XIAP can inhibit hepatic steatosis by suppressing oxidative stress and inflammatory response and impairing NF-E2-related factor 2 activity.^20^ In addition, free fatty acids (FFA) can induce miR-181a-5p expression to inhibit XIAP translation, thereby inducing hepatocyte apoptosis.^21^ However, the effects of XIAP on hepatocyte lipid aggregation and inflammation are unknown.

This study aimed to investigate the biological function of SNHG4 in FFA-induced hepatocyte injury and to reveal its downstream miRNA/mRNA pathways. By investigating the function of SNHG4 and its downstream regulatory axis, this study will contribute to a deeper understanding of the molecular mechanisms of FFA-associated hepatocellular injury, and may provide new biomarkers and therapeutic targets for NAFLD diagnosis and treatment. In addition, the revelation of the complex network of interactions among LncRNAs, miRNAs, and mRNAs will also expand the comprehensive knowledge of the mechanisms of liver diseases.

## Materials and Methods

### Cell Culture

Normal liver cell lines L02 and THLE-2 (Shanghai Yuanchuang Biotechnology Co., Ltd.) were identified by short tandem repeat (STR) analysis. Specifically, 17 commonly used human STR loci were selected for detection, including Amelogenin, CSF1PO, D13S317, D16S539, D18S51, D21S11, D5S818, D7S820, TH01, TPOX, vWA, Penta D, Penta E, D2S1338, D19S433, D3S1358, and FGA. First, genomic DNA from the cells was extracted using a commercial DNA extraction kit to ensure the purity and integrity of the DNA. Then, Promega’s PowerPlex® 18D system was employed to perform multiplex PCR amplification of the selected STR loci, with PCR reaction conditions strictly adhering to the kit’s specifications. Subsequently, capillary electrophoresis on an ABI 3500 Genetic Analyzer was employed to separate the PCR products, with internal standards ensuring accurate fragment size determination. The electrophoresis results were analyzed using GeneMapper® ID-X software to obtain the allelic genotype information for each STR locus. The obtained STR typing results were compared with the reference profiles of the L02 and THLE-2 cell lines in the Chinese STR database of typical human-derived cell lines. The results showed that the cell STR typing was completely consistent with the database profiles of these cell lines, with no cross-contamination or cell misidentification observed. Additionally, during the experiment, negative controls (without DNA template) and positive controls (standard cell line DNA with known STR typing) were included to monitor the specificity and sensitivity of the PCR amplification. Through the above STR analysis, it was confirmed that the cell lines used have authentic and reliable origins and can be used for subsequent experimental studies. Cells were cultured in Dulbecco’s modified Eagle’s medium (DMEM) containing 10% fetal bovine serum (FBS) and kept at 37°C and 5% CO_2_. To establish anin vitro model of NAFLD, L02, and THLE-2 cells were treated with a mixture of FFA (1 mM oleic acid and palmitic acid at a 2:1 in DMEM with 10% FBS) for 24 hours. The study did not involve human or animal testing, so ethical approval and informed consent were not required.

### Cell Transfection

The full-length SNHG4 sequence was cloned into vector pcDNA3.1 (+) (GenePharma). Specific small interfering RNA (siRNAs) of SNHG4 and XIAP, as well as mimic and inhibitor of miR-34b-5p (GenePharma), were designed and synthesized. L02 and THLE-2 cells inoculated into a 6-well plate were transfected using Lipofectamine 3000 (Invitrogen) and tested after 48 hours to validate the transfection efficiency based on real-time PCR (RT-qPCR) or Western blot.

### Oil Red O Staining

Oil red O staining was conducted following the previously described method.^22^ L02 and THLE-2 cells were cultured in a 6-well plate, fixed with 4% paraformaldehyde at 4°C for 15 minutes, and mixed with the oil red O reagent (Sigma, USA) for 15 minutes. The plate was rinsed with distilled water 3 times and imaged under an optical microscope (Zeiss, Germany).

### Metabolometry

According to the manufacturer’s protocol, total cholesterol (TC) and triglycerides (TG) were measured using commercial assay kits (Solarbio, Beijing, China). Cells were seeded in 96-well plates, followed by the addition of extraction buffer for TC detection and a 1:1 mixture of n-heptane and isopropanol for TG detection. The cells were disrupted by ultrasonication on an ice bath. The homogenates underwent centrifugation at 10 000 × g for 10 minutes at 4°C, and the supernatants were retrieved. The absorbance was measured at 500 nm for TC and 420 nm for TG using a microplate reader. The concentrations of TC and TG were subsequently calculated based on their respective calibration curves.

### Enzyme-Linked Immunosorbent Assay

L02 and THLE-2 cells were lysed using radioimmunoprecipitation assay (RIPA) lysis buffer and tested by ELISA kits (Abcam) to determine tumor necrosis factor (TNF)-α, interleukin (IL)-1β, and IL-6 levels. The detection method for the ELISA kit can be referenced on the official website: https://www.abcam.cn.

### Real-Time-Quantitative Polymerase Chain Reaction

Total RNA was obtained from L02 and THLE-2 cells using TRIzol reagent (Shaanxi Dongsheng Biotechnology Co., Ltd.). Ultraviolet (UV) spectroscopy at 260 and 280 nm was used to determine RNA purity and concentration. Complementary DNA (cDNA) was produced by PrimerScript Reverse Transcriptase. The One Step SYBR PrimeScript RT-PCR kit (Takara, Dalian, China) was utilized for RT-qPCR, and the PCR outputs post-amplification were quantified using SYBR Green. The 2^−ΔΔCT^ technique was employed to analyze every target gene, standardizing them against glyceraldehyde-3-phosphate dehydrogenase (GAPDH) or U6. Glyceraldehyde-3-phosphate dehydrogenase and U6 were used as reference genes for L02 cells. Additionally, GAPDH was also used as a reference gene for THLE-2 cells. Primer sequences (Table 1) were commercially synthesized by Sangon.

### Western Blot

Proteins were isolated from L02 and THLE-2 cells utilizing cold RIPA lysis buffer. Protein quantification was performed using a bicinchoninic acid (BCA) protein assay kit from Solarbio. A quantity of 20 μg of protein underwent electrophoresis by 10% sodium dodecyl sulfate-polyacrylamide gel. Then, the protein was moved onto a polyvinylidene fluoride membrane (Millipore, USA), blocked with 5% bovine serum albumin for 40 minutes, followed by an overnight incubation at 4°C with the primary antibody. The membrane was rinsed with 100 μM phosphate-buffered saline with 0.05% Tween-20 and incubated with horseradish peroxidase-linked secondary antibody (1:5000, ab205718, Abcam) for 2 hours. Protein bands were identified with an enhanced chemiluminescence substrate (Thermo Fisher Scientific) and observed with a ChemiDoc XRS+ imager (Bio-Rad). Peroxisome proliferator-activated receptor α (PPARα; sc-398394, Santa Cruz Biotechnology), carnitine palmitoyltransferase 1a (CPT1A; 15184-1-AP, Proteintech), ATP (Adenosine triphosphate)-Binding Cassette A1 (ABCA1; NB400-105, Novus Biologicals), XIAP (610762, BD Biosciences), and GAPDH (60004-1-Ig, Proteintech).

### Bioinformatics Analysis

The potential binding sites between SNHG4, XIAP, and miR-34b-5p were obtained from the “miRNA-LncRNA” and “miRNA-mRNA” sections of the starBase website (https://rnasysu.com/encori/index.php).

### Luciferase Activity Experiment

Network servers predicted the potential binding sites of SNHG4 or XIAP to miR-34b-5p. Wild-type SNHG4 (SNHG4-WT) and XIAP 3’-UTR (XIAP-WT) were subcloned into the psiCHECK2 vector (Promega). The potential binding site mutation (SNHG4/XIAP-MUT) was constructed using the QuickChange II site-specific mutagenesis kit (Agilent). The construct was co-transfected into L02 cells with miR-34b-5p mimic or mimic NC (Negative control) using Lipofectamine 3000 (Invitrogen). Following a 48-hour period, the cells underwent lysis, and their luciferase activity was assessed using the Dual-Luciferase Reporter Assay System (Promega). Luciferase activity was determined by comparing the activity of renilla luciferase with that of firefly luciferase.

### RNA Immunoprecipitation Experiment

RNA immunoprecipitation (RIP) was conducted using the Magna RIP kit (Millipore). L02 and THLE-2 cells, numbering 1 × 10^7^, underwent a cleansing process with chilled PBS (Phosphate buffered saline) and were then gathered using a cell scraper. Cells underwent lysis using RIP lysis buffer (150 mM KCl, 25 mM Tris pH 7.4, 5 mM EDTA, 0.5% NP40, 100 μL per IP [immunoprecipitation] reaction), enriched with mixtures of RNase and protease inhibitors, and then were centrifuged at 12 000 × g for 1 minute at 4°C. After collection, the supernatant was treated with 5 μg of Ago2 antibody (1:1000, 233727; Abcam) or a control IgG (immunoglobulin G) for RNA extraction in PCR. The preparation of the immunoprecipitated magnetic beads adhered to the guidelines provided in the kits. Overnight incubation of the RNA-binding protein-RNA complex occurred at 4°C, accompanied by pre-cleansed magnetic beads and a steady rotation. Following this, Protein G Dynabeads were employed to precipitate the immune complex (40 μL for each IP reaction). Following the bead cleansing using the ice-cold RIP buffer from the Magna RIP kit, proteinase K was introduced and then incubated at 55°C for half an hour to break down RNA-binding proteins. Ultimately, RNA extraction and purification were conducted with TRIzol® reagent, followed by assessing gene expression levels through RT-qPCR.

### Data Analysis

All experiments had at least 3 biological replicates. Data were expressed as mean ± SD. Statistically significant differences were compared and assessed using GraphPad Prism 9.0 (Graphpad Software; Boston, MA, USA) by the two-tailed student t-test (for 2 groups) or one-way analysis of variance (for multiple groups). The significance level was set at *P* < .05.

## Results

### Overexpressing SNHG4 Improves Lipid Metabolic and Inflammatory Disorders Induced by Free Fatty Acids

**Figure 1.**
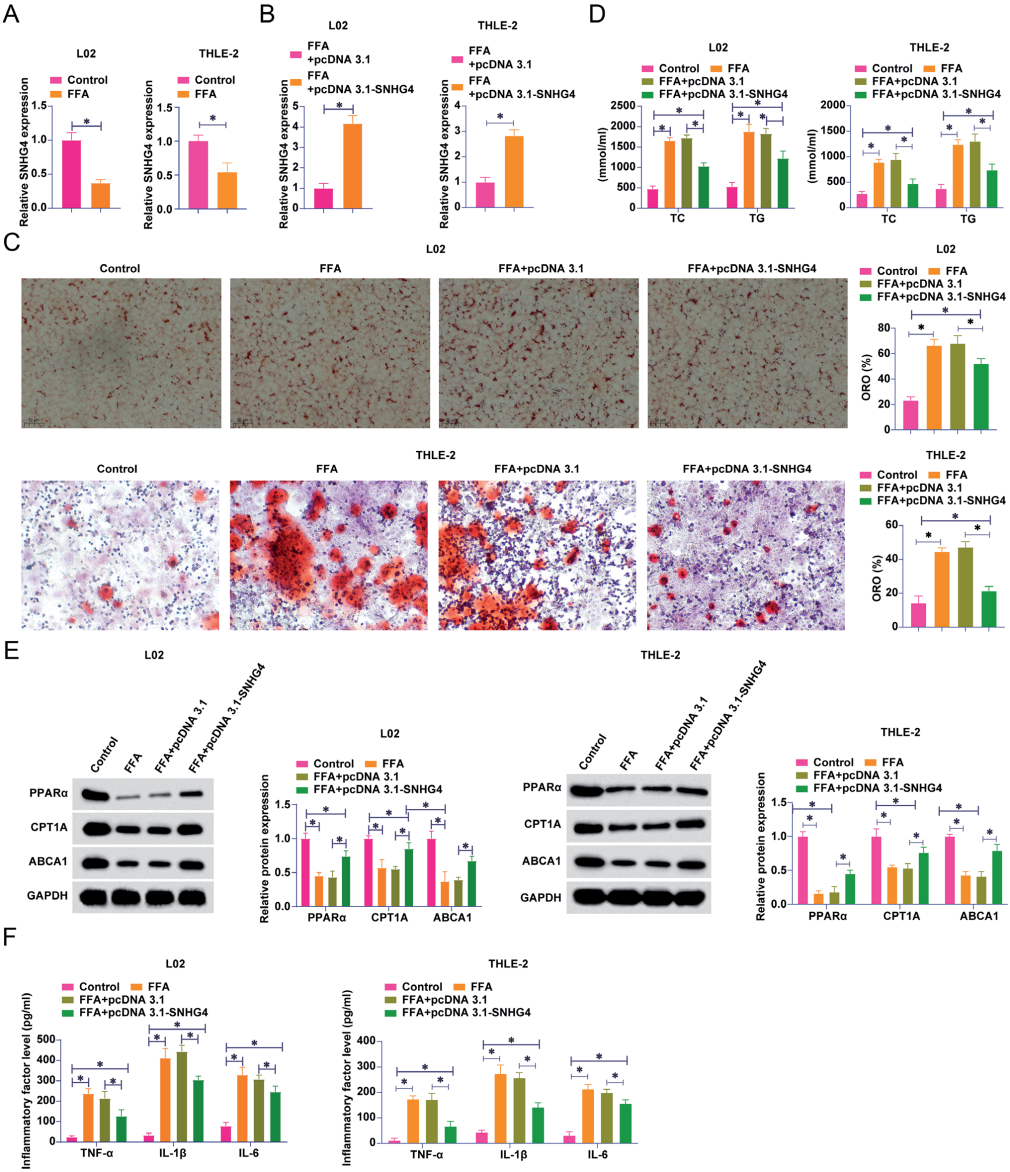
SNHG4 improves lipid metabolism disorder and inflammation induced by FFA. pcDNA 3.1-SNHG4 was transfected into L02 and THLE-2 cells treated with FFA. A-B: RT-qPCR to detect SNHG4; C: Oil red O staining to detect lipid formation; D: Commercial kit to detect TG and TC levels; E: Western blot to measure PPARα, CPT1A and ABCA1; F: ELISA to detect TNF-α, IL-1β and IL-6. Data are expressed as mean ± SD (N = 3). *P < .05.

To explore the biological function of SNHG4 in NAFLD, L02, and THLE-2 cells were treated with FFA to establish an in vitro NAFLD model. FFA treatment induced SNHG4 expression to decrease (Figure 1A). SNHG4-targeting pcDNA 3.1 overexpression vector was transfected into L02 and THLE-2 cells. Figure 1B shows that SNHG4 expression was successfully upregulated. Lipid levels in L02 and THLE-2 cells were assessed by oil red O staining. Free fatty acids treatment promoted lipid formation, while SNHG4 overexpression reduced lipid formation (Figure 1C). Subsequently, lipid formation markers TG and TC were measured. Figure 1D shows that FFA treatment increased TG and TC levels, but this effect was attenuated by overexpressing SNHG4. Subsequently, lipid metabolism-related genes were further evaluated by western blot. Free fatty acids treatment inhibited PPARα, CPT1A, and ABCA1 proteins, but upregulating SNHG4 increased these 3 proteins (Figure 1E). Since chronic inflammation is a main feature of NAFLD, the changes in inflammatory cytokines in cells were analyzed. FFA treatment increased TNF-α, IL-1β, and IL-6 contents, which were inhibited by overexpressing SNHG4 (Figure 1F). These data suggest that SNHG4 expression is reduced in FFA-induced L02 and THLE-2 cells, whereas overexpression of SNHG4 is beneficial in ameliorating FFA-induced lipid metabolism disorders and inflammation.

### SNHG4 Competitively Adsorbs miR-34b-5p

**Figure 2.**
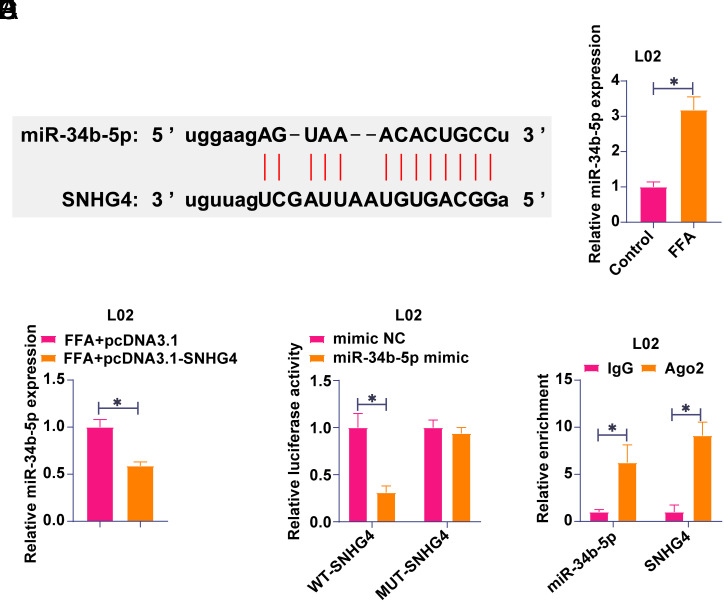
Competitive adsorption of miR-34b-5p by SNHG4. A: Starbase predicted the binding sites of miR-34b-5p and SNHG4; B-C: RT-qPCR to detect miR-34b-5p in L02 cells; D: Dual luciferase reporter assay to examine the targeting relationship between SNHG4 and miR-34b-5p; E: RIP experiment to analyze the binding relationship between SNHG4 and miR-34b-5p; Data are expressed as mean ± SD (N = 3). *P < .05.

Potential downstream miRNAs regulated by SNHG4 were subsequently explored. Starbase, a bioinformatics website, predicted miR-34b-5p and SNHG4 binding sites (Figure 2A). A subsequent study examined miR-34b-5p expression in the in vitro model of NAFLD. Free fatty acid treatment promoted miR-34b-5p expression in L02 cells (Figure 2B) while overexpressing SNHG4 down-regulated miR-34b-5p in L02 cells (Figure 2C). To further validate their relationship, Dual-Luciferase Reporter experiments and RIP experiments were performed. Co-transfection of SNHG4-WT and miR-34b-5p mimic reduced luciferase activity (Figure 2D), and significant enrichment of SNHG4 and miR-34b-5p was found in Ago2 beads (Figure 2E). SNHG4 binds miR-34b-5p competitively, based on these results.

### miR-34b-5p Regulates Lipid Metabolic and Inflammatory Disorders in Non-Alcoholic Fatty Liver Disease by SNHG4

**Figure 3.**
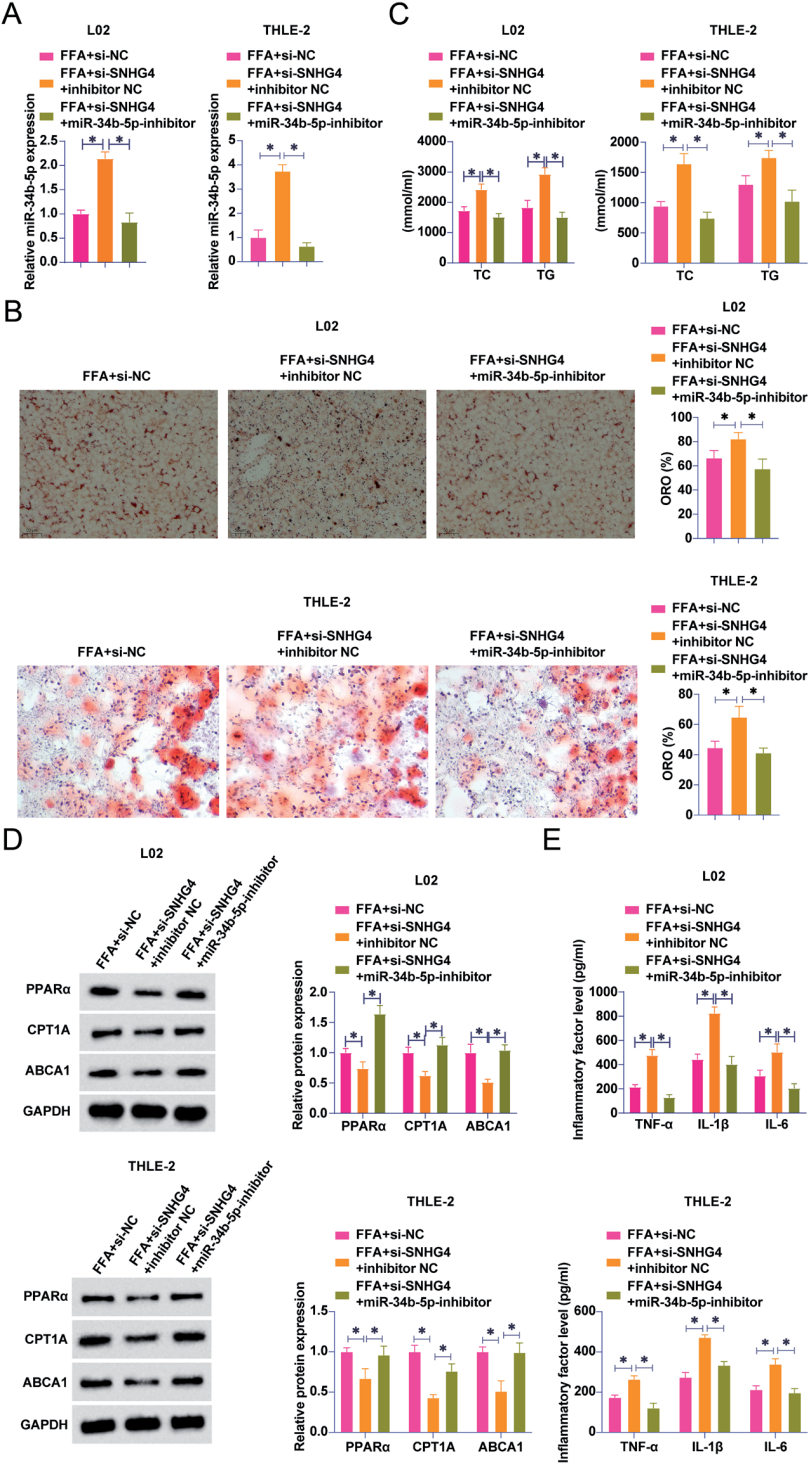
miR-34b-5p is involved in the regulation of lipid metabolism and inflammation in NAFLD by SNHG4. SNHG4-targeting siRNA and miR-34b-5p inhibitor were co-transfected into FFA-treated L02 and THLE-2 cells. A: RT-qPCR to detect miR-34b-5p; B: oil red O staining to detect lipid formation; C: Commercial kit to detect TG and TC levels; D: Western blot to measure PPARα, CPT1A, and ABCA1; E: ELISA to detect TNF-α, IL-1β, and IL-6. Data were expressed as mean ± SD (N = 3). *P < .05.

To investigate whether SNHG4 regulation of NAFLD lipid metabolism and inflammation is related to miR-34b-5p, siRNA targeting SNHG4 and miR-34b-5p inhibitors were co-transfected into FFA-treated L02 and THLE-2 cells. Transfection with si-SNHG4 decreased SNHG4 and increased miR-34b-5p expression, while co-transfection with miR-34b-5p inhibitor decreased miR-34b-5p expression (Figure 3A). Subsequently, the effects of co-transfection on lipid metabolism and inflammation of L02 and THLE-2 cells were analyzed. SNHG4 knockdown increased lipid formation, increased TG and TC levels, inhibited PPARα, CPT1A, and ABCA1 protein expressions, and increased TNF-α, IL-1β, and IL-6 levels; knocking down miR-34b-5p, however, prevented these effects (Figure 3B-E). These data suggest that SNHG4 affects FFA-induced lipid metabolism disorders and inflammation by regulating miR-34b-5p expression.

### X-Linked Inhibitor of Apoptosis Protein is the Downstream Target Gene of miR-34b-5p

**Figure 4.**
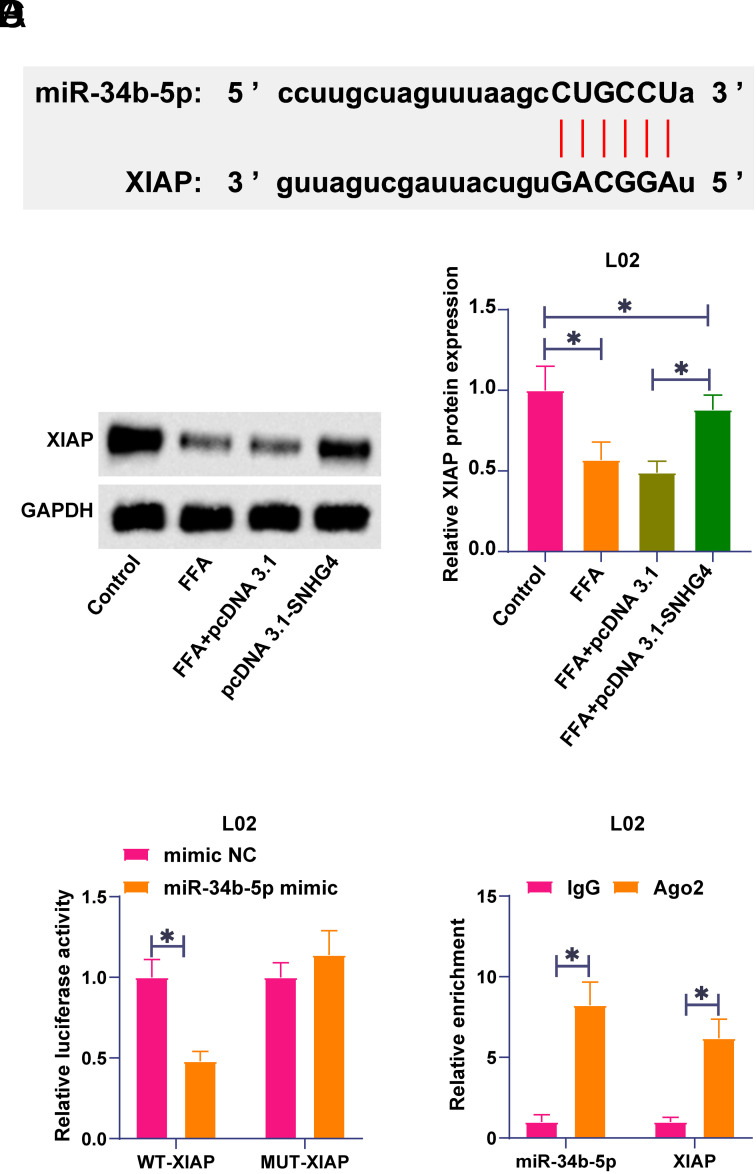
X-linked inhibitor of apoptosis protein is the downstream target gene of miR-34b-5p. A: Starbase predicted the binding sites of miR-34b-5p and XIAP; B: Western blot to measure XIAP in L02 cells; C: Dual luciferase reporter assay to examine the targeting relationship between XIAP and miR-34b-5p; D: RIP experiment to analyze the binding relationship between XIAP and miR-34b-5p. Data were expressed as mean ± SD (N = 3). *P < .05.

Starbase predicted potential binding sites for XIAP and miR-34b-5p (Figure 4A). The expression pattern of XIAP in an in vitro model of NAFLD was analyzed by western blot. FFA treatment inhibited XIAP expression while depleting miR-34b-5p restored XIAP expression (Figure 4B). The dual luciferase reporter experiment and RIP experiment further confirmed their targeting relationship. miR-34b-5p mimic resulted in the reduction of luciferase activity in XIAP-WT (Figure 4C), and XIAP and miR-34b-5p were both enriched in Ago2 beads (Figure 4D), suggesting that XIAP is a downstream target gene of miR-34b-5p.

### SNHG4 Ameliorates Free Fatty Acids-Induced Lipid Metabolism Disorders and Inflammation by miR-34b-5p/XIAP Axis

**Figure 5.**
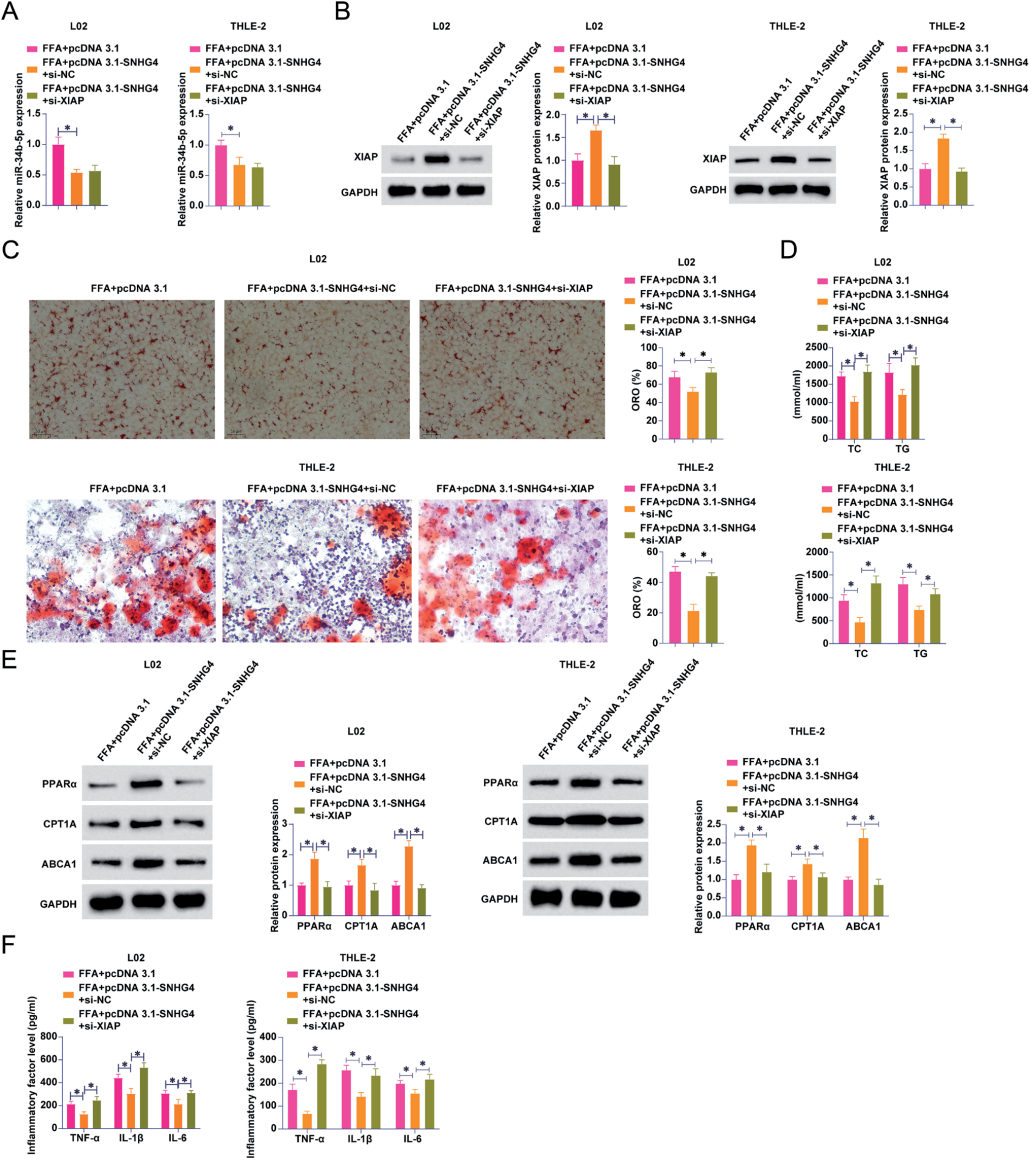
SNHG4 improves FFA-induced lipid metabolism disorders and inflammation by miR-34b-5p/XIAP axis. pcDNA 3.1-SNHG4 and si-XIAP were co-transfected into FFA-treated L02 and THLE-2 cells. A: RT-qPCR to detect miR-34b-5p; B: Western blot to measure XIAP; C: Oil red O staining to detect lipid formation; D: Commercial kit to detect TG and TC levels; E: Western blot to measure PPARα, CPT1A and ABCA1; F: ELISA to detect TNF-α, IL-1β and IL-6. Data were expressed as mean ± SD (N = 3). *P < .05.

pcDNA 3.1-SNHG4 and si-XIAP were co-transfected into FFA-treated L02 and THLE-2 cells. pcDNA 3.1-SNHG4 decreased miR-34b-5p and increased XIAP levels, which were decreased by si-XIAP (Figure 5A and B). Lipid metabolism and inflammation in L02 and THLE-2 cells were subsequently examined. SNHG4 overexpression reduced lipid formation, reduced TG and TC levels, promoted protein expression of PPARα, CPT1A, and ABCA1, and decreased levels of inflammatory cytokines, but these effects were attenuated by knockdown of XIAP (Figure 5C-F). It appears that SNHG4 inhibits FFA-induced inflammation and lipid metabolism disorders by targeting the miR-34b-5p/XIAP axis.

## Discussion

Non-alcoholic fatty liver disease is the most prevalent liver disease in the world, with a global prevalence of 25%.^23^ This study aimed to better understand the lipid metabolism disorders and inflammation associated with NAFLD and ultimately found that SNHG4 ameliorated FFA-induced lipid metabolism disorders and inflammation through the miR-34b-5p/XIAP axis.

Due to increased inflow of FFA and reproduction of liver lipids, NAFLD causes excessive accumulation of TG in hepatocytes.^24^ Regarding this characteristic, FFA treatment has been commonly used to mimic NAFLDin vitro.^25^^,^^26^ In this research, L02 and THLE-2 cells after FFA treatment showed decreased expression of SNHG4, indicating the potential role of SNHG4 in NAFLD. This research mainly investigated NAFLD-associated lipid metabolism and inflammation by determining several indicators. Total cholesterol consists of free cholesterol and cholesterol esters in the blood, which is the sum of all cholesterol contained in lipoproteins in the blood. TC is synthesized in the body by acetyl-CoA, and the liver and small intestine provide about 90% of endogenous TC in adults. Clinically, the measurement of serum TC has become a routine item of lipid analysis. Triglycerides are organic compound that is synthesized from food fat and the liver and are relatively important indicators in lipid analysis. PPARα, a transcription factor activated by ligands, is part of the NR1C nuclear receptor subfamily. Numerous genes targeted by PPARα play a role in the metabolism of fatty acids in rapidly oxidized tissues, like the liver. Activation of PPARα enhances the condition of steatosis, inflammation, and fibrosis in early-stage NAFLD models.^27^ CPT1A is a mitochondrial enzyme that is a rate-limiting step in the oxidation of medium- and long-chain fatty acids beta, producing energy in the form of ATP. Increasing CPT1A activity reduces diet-induced liver TG levels.^28^ In extracellular mediators, ABCA1 transfers cellular phospholipids and free cholesterol to apoA-1 and related proteins.^29^ Moreover, ABCA1 triggers a series of signaling pathways by interacting with apolipoprotein receptors, mediating rate-limiting steps in high-density lipoprotein biogenesis and inflammatory inhibition.^30^ In the measurements of these lipid-associated indicators, it was discovered that SNHG4 suppressed lipid metabolism disorders in FFA-modeled L02 and THLE-2 cells. SNHG4 had anti-inflammatory ability in FFA-modeled L02 and THLE-2 cells by reducing TNF-α, IL-1β, and IL-6 levels. Disease progression is the culmination of a variety of host intrinsic and extrinsic factors that contribute to chronic liver inflammation, which in turn drives NAFLD progression. It has been believed that suppressing inflammation or its source not only reduces liver inflammation and fibrosis but also improves steatosis and metabolic syndrome.^31^ Taken together, SNHG4 protects against FFA-induced lipid metabolic and inflammatory disorders in FFA-treated L02 and THLE-2 cells.

A key role for miR-34b-5p in NAFLD regulation is its involvement in lipid metabolism and inflammation. Experimental analysis revealed that SNHG4 affected FFA-induced lipid metabolism disorders and inflammatory responses by competitive adsorption of miR-34b-5p. Specifically, SNHG4 knockdown resulted in upregulation of miR-34b-5p expression and exacerbated FFA-induced lipid deposition and inflammation, but inhibiting miR-34b-5p reversed these unfavorable effects, suggesting that downregulating miR-34b-5p has a protective effect on lipid metabolism and inflammation in NAFLD models. This is consistent with previous studies in which miR-34b-5p silencing demonstrated protective effects in other inflammatory disease models, such as attenuating injury by inhibiting the release of inflammatory factors in acute lung injury and reducing inflammatory responses in septic renal tubular epithelial cells.^17^ Thus, miR-34b-5p has potential therapeutic target significance not only in NAFLD but also in other inflammation-related diseases. The results indicate that miR-34b-5p may be a therapeutic target in NAFLD and that regulating the SNHG4/miR-34b-5p axis may be an effective way to intervene in pathological processes of NAFLD. In addition, miR-34b-5p silencing has significant anti-inflammatory effects and plays a key protective role in NAFLD by regulating its downstream target gene XIAP. Specifically, miR-34b-5p caused downregulation of XIAP expression in FFA-treated L02 and THLE-2 cells by targeting the inhibition of XIAP, which is closely associated with lipid metabolism disorders and amelioration of inflammation. It was found that XIAP deficiency leads to dysregulation of the TNF receptor signaling pathway, which promotes the manifestation of hyperinflammatory diseases, whereas upregulation of XIAP inhibits NLRP3-associated inflammatory responses and hepatic steatosis triggered by palmitic acid stimulation, thereby alleviating metabolic disorders associated with NAFLD.^20^ Thus, the role of XIAP in NAFLD is not only reflected in the inhibition of inflammation but also in the reduction of lipid accumulation. XIAP plays a significant and complex role in lipid metabolism, with its impact not only dependent on specific cellular contexts but also involving the interplay of multiple biological signaling pathways. In neuronal models, XIAP enhances AMP-activated protein kinase expression and gastrointestinal motility, potentially promoting lipid metabolism indirectly, reflecting its involvement in maintaining cellular energy balance.^32^ In ovarian granulosa cell tumor models, XIAP inhibition alleviates transcriptional repression of PPARγ, thereby restoring PPARγ activity and directly promoting lipid catabolism and metabolic processes, indicating that XIAP influences cell metabolism beyond its anti-apoptotic role.^33^ Notably, in L02 hepatic cells, XIAP regulates miR-34b-5p, affecting key lipid metabolism regulators such as PPARα, while also modulating the interplay between lipid metabolism and inflammation. This suggests that XIAP can influence both lipid metabolism and cellular inflammatory responses simultaneously. Furthermore, the effects of XIAP on lipid metabolism may extend through its regulation of other signaling molecules, potentially impacting the expression and activity of other anti-apoptotic factors and metabolism-associated proteins. This multi-level regulatory mechanism suggests that XIAP is more than just an anti-apoptotic protein; it may interact with various metabolism-related factors to regulate both cell survival and metabolic states. Consequently, XIAP might exhibit dual roles—either promoting or inhibiting lipid metabolism—depending on the specific cellular environment and external stimuli. This functional versatility underscores the crucial role of XIAP in cellular metabolism and highlights its potential as a therapeutic target.

There are several limitations to the study, however, despite finding that SNHG4 modulates the miR-34b-5p/XIAP axis in FFA-induced L02 and THLE-2 cells and alleviates lipid metabolism disorders and inflammation. First, the study was mainly based on the L02 and THLE-2 cell lines, which failed to fully reflect the complex physiological environment* in vivo*, and the lack of validation in animal models and clinical samples limits the clinical translational value of the results. In addition, the study focused on specific regulatory axes and failed to explore other miRNA/mRNA pathways in which SNHG4 may be involved, resulting in an incomplete understanding of its biological functions. Finally, the time- and dose-dependence issues of the experimental design have not been systematically investigated, which may affect the intervention effect. Therefore, in the future, the role of SNHG4 should be further validated in animal models and clinical samples, its generalization to other cell lines should be explored, and its time- and dose-dependence should be systematically investigated. In addition, expanding the exploration of other related pathways and analyzing the expression patterns of SNHG4, miR-34b-5p, and XIAP in NAFLD in conjunction with clinical data will help to reveal a wider range of therapeutic targets and promote the development of SNHG4-related therapeutic strategies, such as gene therapies or small-molecule drugs, with the aim of providing new ideas and means for NAFLD management.

In conclusion, this study provides clues for understanding the mechanism of SNHG4’s role in the field of inflammation and lipid accumulation. Briefly, SNHG4 attenuates lipid metabolism disorders and inflammation in NAFLD by targeting the miR-34b-5p/XIAP axis. Only cellular experiments were performed in this study, which may not completely explain the mechanism of SNHG4/miR-34b-5p/XIAP in NAFLD.

## Figures and Tables

**Table 1.** Polymerase Chain Reaction Primer Sequence

	Primers
lncRNA SNHG4	F: 5′-GATGTCTGACAGCCCTGTGT-3′
	R: 5′-CCCTACCCCCATCTGAGCTAT-3′
miR-124-3p	F: 5′-GCGCAATCACTAACTCCAC-3′
	R: 5′-TGGTGTCGTGGAGTCG-3′
Human	F: 5′-CACCCACTCCTCCACCTTTG-3′
	R: 5′-CCACCACCCTGTTGCTGTAG-3′
U6	F: 5′-CTCGCTTCGGCAGCACA-3′
	R: 5′-AACGCTTCACGAATTTGCGT-3′

## Data Availability

The datasets used and/or analyzed during the present study are available from the corresponding author upon reasonable request.
